# Trigeminal Neuralgia Due to Neurovascular Conflict: A Case Report

**DOI:** 10.7759/cureus.54347

**Published:** 2024-02-17

**Authors:** Sourabh Shinde, Vidya Lohe, Swapnil Mohod, Komal V Dadgal, Unnati Shirbhate, Dhruvi Solanki

**Affiliations:** 1 Department of Oral Medicine and Radiology, Sharad Pawar Dental College and Hospital, Datta Meghe Institute of Higher Education and Research, Wardha, IND; 2 Department of Periodontics, Sharad Pawar Dental College and Hospital, Datta Meghe Institute of Higher Education and Research, Wardha, IND; 3 Department of Pedodontics and Preventive Dentistry, Sharad Pawar Dental College and Hospital, Datta Meghe Institute of Higher Education and Research, Wardha, IND

**Keywords:** gabapentin, carbamazepine, superior cerebellar artery, neurovascular contact, trigeminal neuralgia

## Abstract

A 47-year-old female patient visited the outpatient department with the complaint of “sharp shooting, radiating type of pain” on the maxillary left posterior gingiva for the last three months. The patient was advised a magnetic resonance imaging (MRI) scan which gave the radiological diagnosis of trigeminal neuralgia (TN). It also stated that the root entry zone of cranial nerve-V (CN-V) was in contact with the superior cerebellar artery and anterior inferior cerebellar artery. The patient was kept on a carbamazepine and gabapentin combination and a supportive therapy of multivitamins which brought complete remission within 1.5 months. This case report supports the combination therapy of carbamazepine and gabapentin with supportive therapy of multivitamins.

## Introduction

According to the recent definition given by the third edition of the International Classification of Headache Disorders (ICHD-3) trigeminal neuralgia (TN) is defined as “a disorder characterized by recurrent unilateral brief electric shock-like pains, abrupt in onset and termination, limited to the distribution of one or more divisions of the trigeminal nerve, and triggered by innocuous stimuli” [1]. The pain is often described as “sharp, stabbing or electric shock-like” and the characteristic feature is the “brief pain attacks.” The increased cases of misdiagnoses are seen despite the availability of diagnostic criteria with proper definitions. The pain is often confused with odontogenic pain and results in unnecessary extractions and other dental treatments [2]. The individuals affected are primarily above 50 years with a prevalence of 12 to 30 cases per 1,00,000 people, where women are more frequently affected (60%) than men [1,2] with a ratio of 3:5 . The symptoms are usually described as sudden shooting or stabbing pain, which may be solitary sensations or paroxysms in between pain‐free intervals, which are usually triggered by nonpainful stimuli such as exposure to a gentle breeze, facial touch, brushing the teeth, or chewing food [3]. Single divisions are affected most commonly, but it can affect multiple divisions. In certain cases, the pain is preceded by numbness and a tingling sensation of the face. With time the attacks become more frequent and more severe, whereas certain patients go into remission becoming symptomless for months to years [3]. Certain studies suggest that vitamin B12 plays a crucial role in the demyelination of the nerve which is one of the etiologic factors for TN. As vitamin B12 is a methyl donor and plays a crucial role in the maintenance of myelin sheath [4].

The historical evidence date the literature as early as the second century A.D. in the literature of Aretaeus of Cappadocia, despite having such early roots, it took the 18th century to form the first published resource of TN by John Fothergill (1773), hence also referred to by some as Fothergill’s disease [5]. It is classified as idiopathic, neuropathic and deafferentation, symptomatic and postherpetic, and atypical facial pain. The idiopathic type, which is further subclassified as TN type 1 (TN 1) is classical or typical, and TN type 2 (TN 2) is “idiopathic trigeminal facial pain” that presents as aching, throbbing, or burning. The type of pain in neuropathic and deafferentation is present anywhere along the path of the nerve, and the patient presents with a history of injury to the nerve. The pain is neuropathic as opposed to the idiopathic forms [5]. Symptomatic and postherpetic type of TN is associated with cases of demyelination of the nerve, and often the TN in these cases is associated with multiple sclerosis. It presents with either an episodic or constant nature of pain. In patients with atypical facial pain, the patients present with pain which spreads outside of trigeminal distribution [5].

## Case presentation

A female patient aged 47 years old reported to the outpatient department with the chief complaint of “sharpshooting, radiating type of pain” on the maxillary posterior gingiva region on the left side in the past three months. There were intermittent, short-lasting episodes of pain which were severe mainly affecting the maxillary posterior region on the left side. The pain was radiating to the upper lip, maxillary sinus, and nostril region on the left side, the area covered by the maxillary branch. The patient also described it as an “episodic electric shock-like feeling after the initiation of the pain.” The provoking factors controlled the frequency. The patient gave a history of 20-25 episodes a day, which were provoked by light touching, drinking water, rinsing, and brushing. The visual analogue scale (VAS) assessment gave a score of 8-9. The patient had a history of extractions of 26 and 27 two months prior in the same region, where the patient gave a history of the same type of pain and the pain did not subside even after repeated extractions. The medical history was not significant. The patient did not give any history of adverse habits and was not allergic to any medications known to her to date.

**Figure 1:**
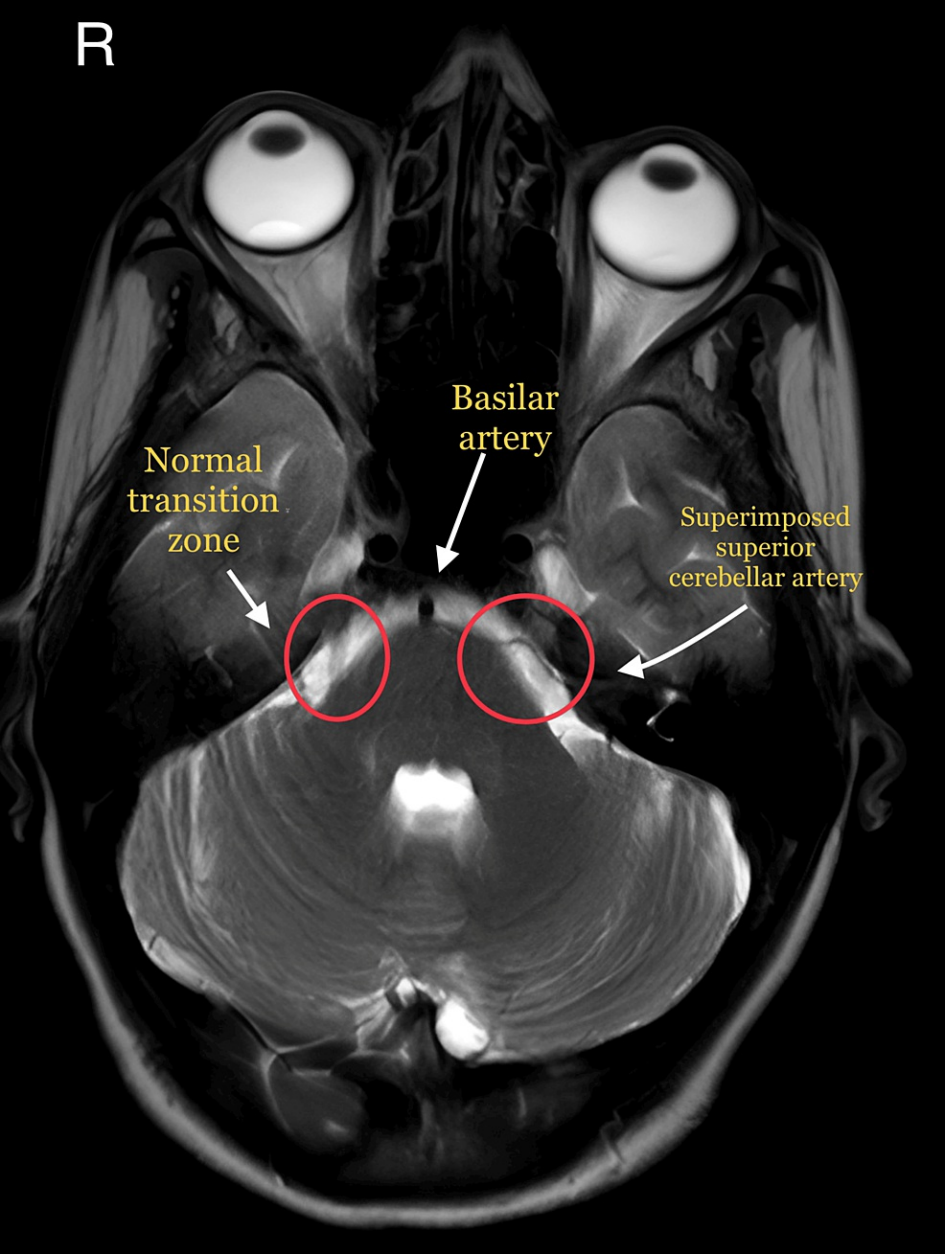
The left transition zone is "sandwiched" between superior cerebellar artery and anterior inferior cerebellar artery

**Figure 2:**
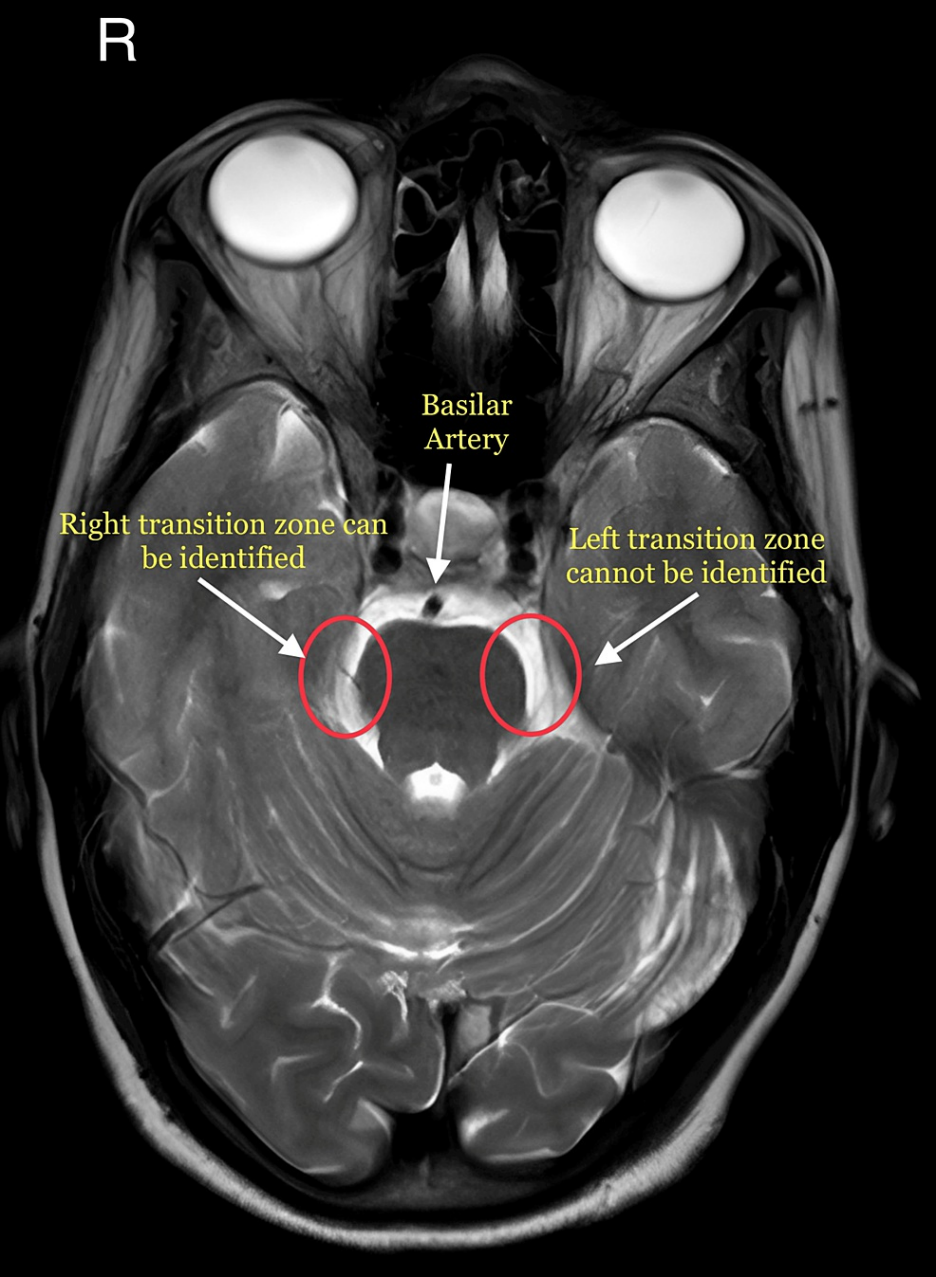
The left transition zone cannot be located

On examination, the facial features were symmetrical. There were no skin discolorations, masses, or lesions that were observed in the head and neck region. The lips were competent and “touching the left upper lip aggravated the pain episode that the patient confirmed as the type of pain that she had been experiencing for the last two months.” On palpating the temporomandibular joint and associated muscles, patient did not report any pain. On palpation, there was no regional lymphadenopathy. On intraoral examination, while gently touching the gingiva on the maxillary left posterior region, the patient confirmed a similar episode as experienced before, and therefore, the patient was asked to rest till the remission of the episode. The teeth in the vicinity were asymptomatic. After observing the above features, the provisional diagnosis was given as TN of the left maxillary branch. As part of the investigations, the patient was advised a magnetic resonance imaging (MRI) scan for further evaluation. The findings of the MRI showed that the “transition zone of the left trigeminal nerve was sandwiched between left superior cerebellar artery and anterior inferior cerebellar artery" as shown in Figure 1 and Figure 2. 

Otherwise, there was “no obvious abnormality in the brain parenchyma.” After the radiographic diagnosis, the patient was kept on a tablet of carbamazepine 600 mg divided into three equal doses daily as the primary treatment and with multivitamin tablets as a supportive therapy. The regimen was advised for seven days. On the second visit, the patient did give a history of reduction in the number of episodes from 20-25 to 18-20 episodes, and the severity of pain did reduce as the patient gave a VAS score of 7-8. The patient was advised to continue the same regimen of medications for another seven days and recalled after seven days. On the third visit, patient again gave the history of reduction in the number of episodes to 14-15 episodes a day and reduced the severity of pain with a VAS score of 6-7. Hence, as there was no significant remission seen, addition of tablet gabapentin 100 mg with the original regimen was advised for seven days, and a recall after seven days was given. On the fourth visit, the patient gave a history of reduced episodes to 10-11 episodes per day and reduced severity of pain with a VAS score of 4-5.

The patient was continued on the same medications. The patient was recalled after seven days. On the fifth visit, the patient gave a history of 5-6 episodes daily and a VAS score of 2-3. The patient was again recalled after seven days. On the sixth visit, the patient gave a history of complete remission of pain. To this day, the patient is pain-free. 

## Discussion

The “neurovascular conflict” in the prepontine cistern is the primary pathophysiological cause of TN, which often results in the “compression” of the cranial nerve-V (CN-V) by a blood vessel at the “root entry zone” [1]. Another reason for TN is the cavernous malformations. They are vascular lesions that are “occult” and present an incidence of 0.3% to 0.5% and present as “iterative hemorrhagic episodes, site-specific signs and symptoms, and seizures” [6]. Patients with secondary TN present with pathologic changes like tumors (meningiomas, neuromas, and cysts). Here, in this case, we can see that there was no pathology involved, and the cause was purely anatomical. Cases of aneurysms or arteriovenous malformations may also present with secondary TN, but they are less frequent [7]. TN despite having typical pain still confuses the patient as well as clinicians with the odontogenic pain. This results in needless extractions and pulp therapies in the teeth of the involved regions [8]. This case was no excuse for misdiagnosis as the patient reported extractions of teeth two months back. MRI remains the gold standard for the diagnosis of the condition as the etiologic considerations involve both hard and soft tissue components as well as the vascular component. The most important aspect of the MRI is that it gives a clearer view of the root entry zone [9]. In this patient, MRI played a crucial role as it ruled out the pathological causes and confirmed the indentation of the superior cerebellar artery over the trigeminal nerve.

Carbamazepine has a long supportive literature to be the drug of choice, and the doses vary from 100 mg/day to 2400 mg/day. The patient most probably responds to 200 mg/day to 800 mg/day. If the patient shows sensitivity to carbamazepine, then “oxcarbazepine” should be administered at 200 mg/day to 2400 mg/day in two to three divided doses [10]. If the patient is unresponsive to the medicinal approach, then the patient should be advised for surgery. Vitamin B12 therapy should be given as an adjunct therapy as it plays a crucial role in maintaining the integrity of the myelin sheath and also helps in the repair of the myelin sheath if it is damaged [4]. In surgical procedures, microvascular decompression can be considered the gold standard treatment for TN. It is often indicated in patients with diagnosed neurovascular conflict, but it is recommended for patients who can withstand open neurosurgical procedures. Other treatments include "endoscope-assisted microvascular decompression," "percutaneous balloon decompression," and "percutaneous glycerol injection," and "extracranial peripheral denervation" [11]. Radiosurgery procedures mainly "gamma knife radiosurgery" and "radiofrequency rhizotomy" are among the recent advances where the fields of radiology and neurosurgery are merged to form a minimally invasive form of surgical treatment modality [12]. In gamma knife radiosurgery, a median dose of 80 Gy is targeted onto the involved region with a 4 mm collimator. It is performed in cases of malformation of the nerve (e.g., atrophy) [12].

## Conclusions

TN is a condition that majorly affects the day-to-day activities and mental health of the patient. The key challenge that is presented with the disorder is the misdiagnosis and needless odontogenic treatments. Patients usually drain themselves mentally due to repeated unnecessary treatments that might be invasive in nature with negative remission of pain. Hence, a proper diagnosis is a must, and if possible, an early diagnosis should be made. The treatment protocol should be thorough, and patient counseling should be done regarding the same.

## References

[1] Silva J, Viguini Tolentino Correa A, Alves da Silva I, Silva Pinto de Carvalho C, Ramina R (2023). Trigeminal neuralgia due to unilateral early bifurcated superior cerebellar artery: case report and literature review. Cureus.

[2] Latorre G, González-García N, García-Ull J (2023). Diagnosis and treatment of trigeminal neuralgia: consensus statement from the Spanish Society of Neurology's Headache Study Group. Neurologia (Engl Ed).

[3] Turton M, Malan-Roux P. (2019). Trigeminal neuralgia: case report and literature review. Stomatological Dis Sci.

[4] Dhole P, Lohe V, Bhowate R, Gondivkar SM, Kadu R, Mohod SC, Sune RV (2022). Evaluation of serum vitamin B12 levels and its correlation with clinical presentation in patients with trigeminal neuralgia. J Oral Biol Craniofac Res.

[5] Eller JL, Raslan AM, Burchiel KJ (2005). Trigeminal neuralgia: definition and classification. Neurosurg Focus.

[6] Hegarty AM, Zakrzewska JM (2011). Differential diagnosis for orofacial pain, including sinusitis, TMD, trigeminal neuralgia. Dent Update.

[7] Cao C, Li M, Wu M, Jiang X (2023). Trigeminal neuralgia secondary to osteoma and vascular compression: illustrative case. J Neurosurg Case Lessons.

[8] Deshmukh VR, Hott JS, Tabrizi P, Nakaji P, Feiz-Erfan I, Spetzler RF. (2005). Cavernous malformation of the trigeminal nerve manifesting with trigeminal neuralgia: case report. Neurosurgery.

[9] Bora N, Parihar P, Raj N, Shetty N, Nunna B (2023). A systematic review of the role of magnetic resonance imaging in the diagnosis and detection of neurovascular conflict in patients with trigeminal neuralgia. Cureus.

[10] Oliveira CM, Baaklini LG, Issy AM, Sakata RK (2009). Bilateral trigeminal neuralgia. Case report. Rev Bras Anestesiol.

[11] Rapisarda A, Battistelli M, Izzo A (2023). Outcome comparison of drug-resistant trigeminal neuralgia surgical treatments-an umbrella review of meta-analyses and systematic reviews. Brain Sci.

[12] Iwai Y, Ishibashi K, Yamanaka K (2021). Gamma knife radiosurgery for concurrent trigeminal neuralgia and glossopharyngeal neuralgia. Cureus.

